# Monitoring Street-Level Spatial-Temporal Variations of Carbon Monoxide in Urban Settings Using a Wireless Sensor Network (WSN) Framework 

**DOI:** 10.3390/ijerph10126380

**Published:** 2013-11-27

**Authors:** Tzai-Hung Wen, Joe-Air Jiang, Chih-Hong Sun, Jehn-Yih Juang, Tzu-Shiang Lin

**Affiliations:** 1 Department of Geography, National Taiwan University, No. 1, Sec. 4, Roosevelt Road, Taipei 10617, Taiwan; E-Mails: chsun@ntu.edu.tw (C.-H.S.); jjuang@ntu.edu.tw (J.-Y.J.); 2 Department of Bio-Industrial Mechatronics Engineering, National Taiwan University, No. 1, Sec. 4, Roosevelt Road, Taipei 10617, Taiwan; E-Mails: jajiang@ntu.edu.tw (J.-A.J.); d98631001@ntu.edu.tw (T.-S.L.); 3 Taiwan Geographic Information System Center, No. 1, Sec. 1, Roosevelt Road, Taipei 10066, Taiwan

**Keywords:** wireless sensor network (WSN), street-level air quality, real-time monitoring, geographic information system (GIS)

## Abstract

Air pollution has become a severe environmental problem due to urbanization and heavy traffic. Monitoring street-level air quality is an important issue, but most official monitoring stations are installed to monitor large-scale air quality conditions, and their limited spatial resolution cannot reflect the detailed variations in air quality that may be induced by traffic jams. By deploying wireless sensors on crossroads and main roads, this study established a pilot framework for a wireless sensor network (WSN)-based real-time monitoring system to understand street-level spatial-temporal changes of carbon monoxide (CO) in urban settings. The system consists of two major components. The first component is the deployment of wireless sensors. We deployed 44 sensor nodes, 40 transmitter nodes and four gateway nodes in this study. Each sensor node includes a signal processing module, a CO sensor and a wireless communication module. In order to capture realistic human exposure to traffic pollutants, all sensors were deployed at a height of 1.5 m on lampposts and traffic signs. The study area covers a total length of 1.5 km of Keelung Road in Taipei City. The other component is a map-based monitoring platform for sensor data visualization and manipulation in time and space. Using intensive real-time street-level monitoring framework, we compared the spatial-temporal patterns of air pollution in different time periods. Our results capture four CO concentration peaks throughout the day at the location, which was located along an arterial and nearby traffic sign. The hourly average could reach 5.3 ppm from 5:00 pm to 7:00 pm due to the traffic congestion. The proposed WSN-based framework captures detailed ground information and potential risk of human exposure to traffic-related air pollution. It also provides street-level insights into real-time monitoring for further early warning of air pollution and urban environmental management.

## 1. Introduction

A well-established air quality monitoring system can enhance our understanding of the quality of life of the built environment. Empirical relationships between air pollutants and human health can be further discovered by combining the data of air quality and health outcomes [1]. Early warning thresholds and environmental exposure health risks can be assessed using these empirical relationships. Therefore, many studies on air quality monitoring employ precise but expensive instruments to measure variations of air pollution on a large scale, such as over a city or even a county [2,3]. Most of the data of these studies were from government or academic institutes that established limited monitoring stations in a large-scale region to collect overall air pollutant conditions. For instance, the Environmental Protection Administration (EPA) of Taiwan deployed thirteen stations to monitor the air quality of Taipei City over an area of 272 km^2^. In other words, each sensor in a station was responsible for monitoring almost 2,600 football fields with a total size of 20 km^2^. The fine-scale geographic heterogeneity of air quality cannot be identified simply by measuring the overall patterns of a large-scale region. Therefore, traditional approaches limit the possibility of exploring the fine-scale spatial-temporal variations of air pollutants in the street-level environment and exposure to air pollution during traffic congestion.

Establishing a street-level monitoring system of air quality can assist in exploring fine-scale relationships between air pollutants and human activities. The sensors of a street-level monitoring system can capture fine-scale spatial-temporal variations of air quality. The information can help pubic administrators gain a more realistic picture of quality of life. In recent years, with the rapid growth of the manufacturing process in semi-conductor technology, such as smaller chip sizes and new sensing materials, lower-power consumption and better measurement accuracy can be achieved simultaneously [4,5]. It is now possible to deploy an effective wireless sensor network (WSN) in urban settings for studies on environmental monitoring [6].

A WSN consists of spatially distributed sensors to monitor environmental conditions, such as temperature, noise and air pollution. Sensors in a WSN cooperatively pass their data over the wireless network to the main database. The growing body of literature focuses on the following topics. An optimized method to employ the fewest sensors to monitor a study area while obtaining accurate results has been studied by Al Hasan *et al.* [7]. Several authors have studied sensor technologies with lower power consumption to extend WSN’s application to transportation and environmental management fields [8,9,10,11]. Most studies on wireless sensor networks have focused on technical aspects, such as network design or data protocols [12,13]. Several studies have applied WSNs to environmental monitoring focused on the dynamics of indoor air pollutants. Green *et al.* explored the *spatial-temporal* variation of temperature and oxygen concentrations inside silage stacks [14]. Aida deployed micro-sensors to form a smart office [15]. Murty and his colleagues designed a sensor network to detect a sodium leak in a nuclear power plant [16]. Applications in the outdoor environment have focused on monitoring agricultural settings such as crops [17,18] or aquaculture, such as sea cucumbers [19].

The exposure risk to humans in outdoor environments is also an important issue. Jung *et al.* designed an air pollution monitoring system using a geo-sensor network that involves a context model and a flexible data acquisition policy to form a guideline to save limited batteries because it reduces the amount of data transmitted [20]. Khedo *et al.* also planted numerous sensors to form a wireless sensor network air pollution monitoring system (WAPMS) in Reduit, Mauritius, to estimate the air quality index (AQI) of ozone (O_3_), fine particulate matter, nitrogen dioxide (NO_2_), carbon monoxide (CO), sulfur dioxide (SO_2_) and total reduced sulfur compounds over the whole island [21]. The study focused on the algorithm used to reduce the amount of data to be transmitted to save energy but not the possibilities of the monitoring results. Ma *et al.* presented an air pollution monitoring and mining system based on a wireless sensor network and grid computing [6]. A low-cost sensor network to collect real-time large-scale data from road traffic emissions was established to monitor air pollutants, such as benzene (C_6_H_6_), NO_2_ and SO_2_ in the built environment. The study considered road networks and traffic flow but only focused on the networking performance. Resch *et al.* proposed the concept of “Live Geography”, which means using communication technology of geo-sensors to monitor real-time dynamics of environmental and social phenomena in geographical spaces [22]. They used CitySense sensor network to build the mobile sensing environment in Cambridge and the city of Copenhagen. The study developed an interoperable open standards based infrastructure for providing air quality data. It also allows users to assess real-time environmental conditions.

Great advances in system frameworks, infrastructures and hardware of sensor network have been achieved in previous studies; however, research on exploring and comparing the street-level spatial-temporal patterns of air quality using wireless geo-sensors is still limited. Monitoring the variations of street-level air pollution can reflect short-term high concentrations of traffic-related air pollutants, including sulfur oxides, ozone, carbon oxides and suspended particles [23]. CO is emitted by incomplete combustion in automobile engines [24,25]. Hence, a rising CO concentration can reflect daily heavy traffic periods and localized traffic areas. Therefore, the purpose of this study is to propose a pilot framework for a WSN-based environmental monitoring system of air pollution to estimate the exposure risk to humans. Because carbon monoxide (CO) is the major traffic pollutant from automobiles, the system further measures real-time fluctuations and analyzes street-level spatial-temporal changes of CO for urban environmental management policy.

## 2. Data and Method

### 2.1. Study Area

The WSN was implemented in the Da-an District, Taipei City, in 2010. Taipei is the capital of Taiwan and hosts the highest population density (9,800 persons/per km^2^ on average) [26]. The city is classified as humid subtropical climate conditions based on the most popular Köppen climate classification, and is strongly influenced by the East Asian monsoon. According to the long-term climate data from Central Weather Bureau of Taiwan, the annual mean temperature in Taipei is 23.0 °C ranging from the lowest monthly mean of 16.1 °C in January to the highest monthly mean of 29.3 °C in July. The city is also a humid region with the annual mean relative humidity (RH) of 76.6 % and the annual precipitation of 2,405.1 mm. The Da-an District is an important educational, commercial, residential, and cultural center of Taipei City with a high population density of 27,500 living within a square kilometer. The dense population facilitates a significant effect of human activities on the local environment, such as traffic congestion near arterials that produce air quality fluctuations within a small area. One major source of air pollution in Taipei is from mobile emission sources, particularly motorcycles. This study deployed a wireless sensor network with a length of 1.5 km. The network was installed along an arterial (and a circle)-connected residential area in satellite cities with the central business district of Taipei. 

### 2.2. WSN-Based Framework

**Figure 1:**
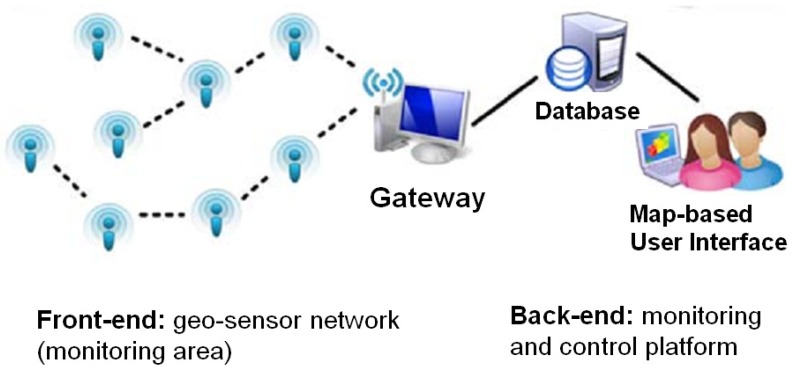
System overview: The wireless sensor network comprises two major components: (**1**) front-end: geo-sensor network and (**2**) back-end: monitoring and control platform. The sensor network is responsible for measuring the concentration of air pollutants in the study area. The monitoring and control platform receives monitoring data through a map-based system to provide real-time information of the sensor data. The data gateway nodes connect the front end and back end by wireless Internet.

The WSN was composed of two major components as, shown in Figure 1: (1) sensor nodes that collected real-time air quality at the street-level scale, with each sensor node including a signal process module, a carbon monoxide (CO) sensor, and a wireless communication module; and (2) a map-based monitoring and control platform for the visualization and management of sensor data. The detailed system framework and monitoring process are described as follows.

#### 2.2.1. Deployment of Wireless Sensors

Our WSN was composed of various types of sensor nodes, including air quality sensors, transmitter nodes and gateway nodes. Each air quality sensor (a total of 44 sensors in this study) was composed of a carbon monoxide sensing module and a wireless module. Forty transmitter nodes were used to support island hopping of the monitoring data. The average distance between sensors was 46.2 m, which means the monitored results were not transferred directly to the main database but sensor to sensor. Four gateway nodes with a weather module acted as a transmission station from the sensor nodes to the database. The WSN was implemented to monitor street-level air quality from July to September 2010.

**Figure 2:**
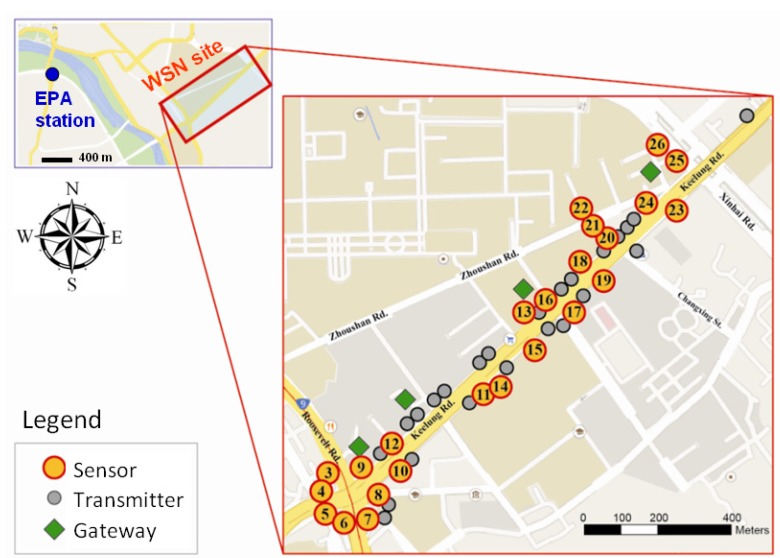
Study area: The wireless sensor network was distributed along an arterial connecting a residential area and the central business district and a circle intersecting another arterial connecting the south and north of the city. The yellow dots present the air pollutant sensors, the grey dots symbolize the transmitters and the green diamonds are the data gateway nodes of the wireless sensor network.

Because of the complex building structures of the built environment, we tested the efficiency of the data transmission and we considered environmental settings (e.g., obstructions) to determine where to install the sensors in the initial stage. The transmitter nodes play an important role in the obstacle-crossing transmission of the signals. Previous studies employed a reticular structure to enhance data transmission and reduce power consumption. However, the WSN used for monitoring traffic air pollutants was restricted to the linear structures of roads. As depicted in Figure 2, we also considered the density of street trees. The left side of the arterial has more sensor nodes because it has more street trees to obstruct signal transmission. A series of signal analyses were implemented to calibrate the reliability and stability of the wireless network. Thresholds were set that the wireless signal strength should be greater than −80 dBm and the packet reception rate should exceed 90% to ensure stable transmission from sensor to sensor. The maximal spacing between sensor nodes is 50 m and the wireless sensor sampling interval is 10 seconds. The transmission quality of the signal strength remained at −72.5 dBm, and the reception rate was 94.32% on average. The average distance between sensor nodes was 46.2 m. Furthermore, to simulate realistic human exposure to traffic pollutants, all sensors were deployed at a height of 1.5 m on lampposts and traffic signs, representing the distance from the ground to the nose of an adult. The sensors were also located near traffic signs, bus waiting zones and intersections of the main roads to explore the spatial-temporal variations of CO concentrations produced by traffic flows.

**Figure 3:**
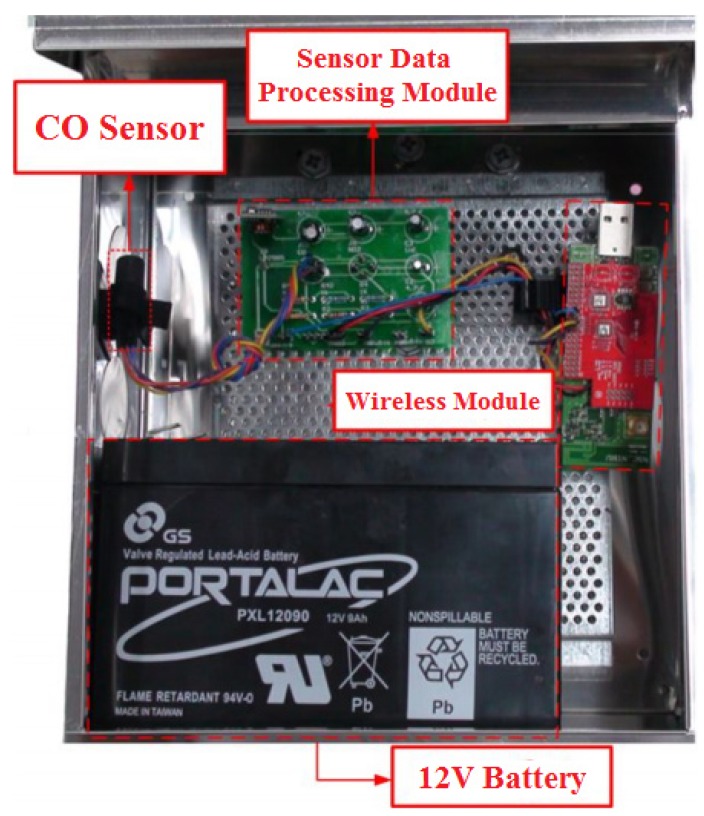
Instruments: A sensor node includes a semi-conductor carbon monoxide sensor, a sensor data processing module and a wireless module. The semi-conductor sensor is used to measure the concentration of air pollutants with variations of voltage. The processing module transfer the voltages gathered by the CO sensor to the CO concentration. The wireless module transmits the monitoring data among sensor, transmitter, and gateway nodes. A 12-V lead-acid rechargeable battery was used to provide system power.

This study employed ZigBee as the communication protocol of the wireless system framework. ZigBee uses small, low-power radios based on the IEEE 802.15.4 standard for data transmission and is for applications with low transmission rates and a long battery life. Therefore, ZigBee is suitable for the long-term monitoring of air pollutants [27,28]. The sensor is housed in a 6,900 cm^3^ steel container as shown in Figure 3. A MiCS-5525 metal oxide semiconductor CO sensor from e2V was selected due to its small size and reasonable accuracy. The detection range of the CO sensor is from 1 to 1,000 ppm and its sensing resistance in air is 100–1,500 kΩ with the air conditions at 23 ± 5 °C and 50 ± 10% RH . The MiCS-5525 is equipped with its own activated carbon filter to minimize the possible interruption errors. This sensor captures the conductivity of the sensing layer, made of a metal oxide semiconductor, such as SnO_2_ or ZnO, to measure the CO concentration. Each sensor consumes only 168 mW when idle and 371 mW when working, therefore providing a more dense sampling frequency. Each sensor station was supplied by a 12-volt lead-acid rechargeable battery. The battery needed to be changed every week to sustain data continuity and signal strength.

The gateway nodes collected the monitoring data from the sensor nodes and sent them to the database by Wi-Fi, thus saving huge compared with other protocols that charge users according to the volume of transmission. An ARK-3360L industrial computer from Advantech was selected for its high resistance to temperature, moisture and impacts. 

#### 2.2.2. Concentration of Carbon Monoxide (CO)

**Figure 4:**
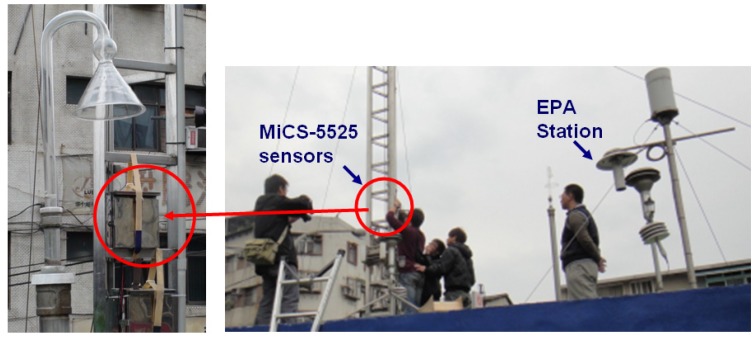
Settings of MiCS-5525 sensors at an EPA station for calibrating CO concentration before deploying wireless sensors.

The MiCS-5525 (e2V) sensors monitored the ambient CO concentration through the fluctuations of conductivity on sensing layer. To calibrate the readings of CO concentration of the sensors, before deploying CO wireless sensors, we placed every sensor used in this study at an EPA station to establish the calibration function for each sensor (Figure 4). Under similar environmental conditions, the CO concentrations measured by EPA were treated as the references to link the voltage readings of the MiCS-5525CO sensors. We then used scatter plots to build the relationship between the readings of CO concentrations from EPA and voltage readings of MiCS-5525 sensors.

**Figure 5:**
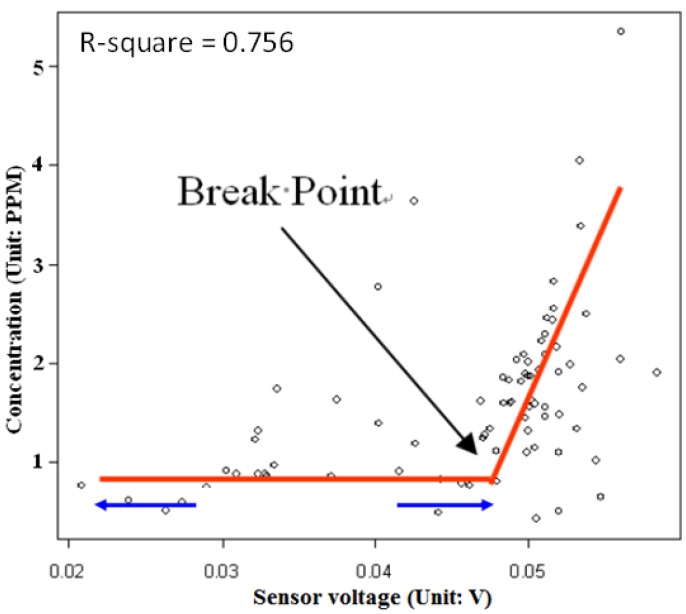
Segmented regression was employed to transfer the voltage of the sensor to the concentration of carbon monoxide. Every sensor has its own regression equation. The method identifies a break-point to separate the regression model into two parts: the flatter trend line represents no association, and the steeper one explains the linear relationship between the concentration and the sensor voltage.

As shown in Figure 5, the relationship between the two factors is non-linear. There was a lower bound because the minimum detection limit of the MiCS-5525 CO sensor is 1 ppm. To avoid inducing a possible bias in concentration estimation, we employed segmented linear regression to form a break point in the regression estimation to build a linear relationship between voltage and concentrations above the threshold. 

Segmented linear regression uses a break point to achieve a better fitting of the model, especially when two linear relationships between independent and dependent variables co-exist. The regression results revealed that 90% of the above sensors had thresholds to predict concentrations from voltages. When the sensors captured voltages from 0.03 to 0.05, the linear relationship was stable. A voltage above 0.06 did not produce a reliable relationship between voltage and concentration. Based on the different micro-environment sensor locations, this study allowed each sensor to have a specific regression model. Therefore, there are 44 equations of air pollution concentration in this study, with an average 0.741 r-square value. 

#### 2.2.3. Map-Based Monitoring and Control Platform

**Figure 6:**
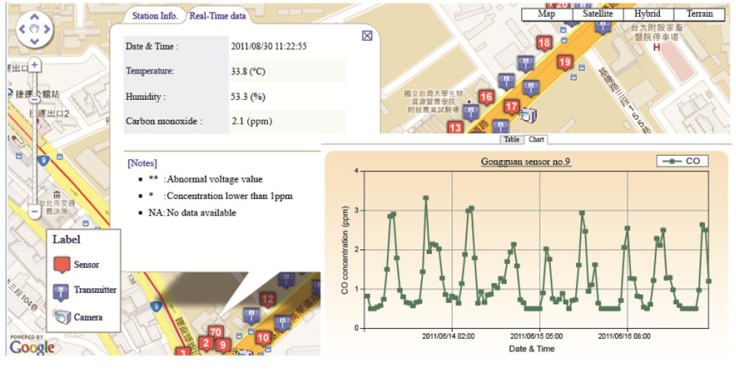
Map-based monitoring and control platform.

The map-based monitoring and control platform was composed of three components. First, an information-processing system was employed to integrate and transform air quality data from multiple sources. Second, the vehicle flow data were combined. Third, the web notification service was also integrated to share air quality information flexibly. A multiple-source monitoring platform was also established. In addition to the WSN-based data we deployed, the platform also received air quality data from the environmental authorities of the central and local governments. The platform then unified the data format and published the monitoring results through website http://www.wsn.org.tw:8080/wsn/. Google Maps and bar charts were used to present the real-time information of the street-level air quality. The trend graph shows the historical information stored in the geographic information system (GIS) to enhance understanding of the spatial-temporal changes (Figure 6). It can be observed that the real-time concentration was obtained with its historical data to show a significant fluctuation between the on/off peaks of traffic. The sensors from the different platforms also provided real-time monitoring results. It is possible to aggregate the monitoring results to temporal resolutions such as hourly, daily and weekly. Therefore, the trend of the CO concentration can be presented with different temporal resolutions. 

## 3. Results and Discussion

### 3.1. Mean of Hourly Concentrations

**Figure 7:**
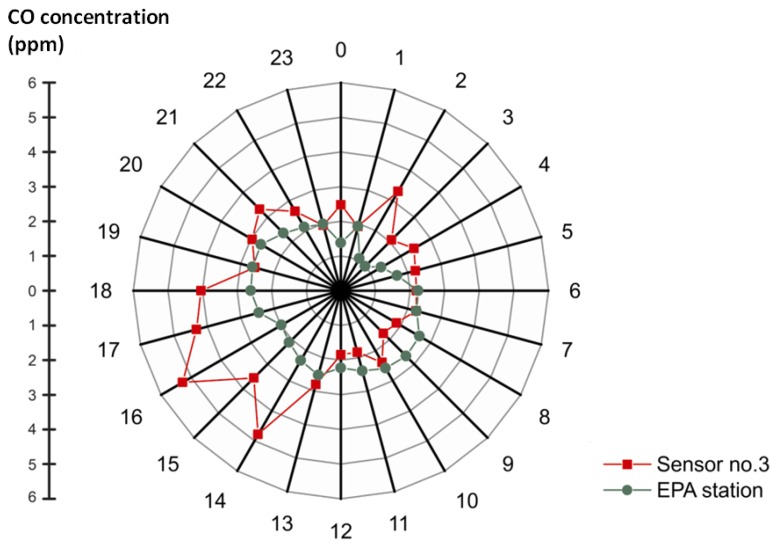
Comparisons of hourly average CO concentration between Sensor No.3 and the EPA station over different time of a day from July to September 2010.

We compared the differences of the hourly CO concentrations from the WSN-based sensor with the EPA monitoring station in similar urban settings. Figure 7 depicts the hourly average CO concentration from July to September 2010 at sensor No. 3, which was located along an arterial (Keelung Road), and the distance to the nearest traffic sign was approximately 15 m. Four carbon monoxide (CO) concentration peaks were distributed throughout the day: (a) the peak at 2:00 pm, with an average concentration of 4.52 ppm, was due to lunch traffic during mid-day; (b) the highest peak was from 5:00 pm to 7:00 pm, with an average concentration of 5.3 ppm, due to congested traffic related to the off-duty time; (c) the peak at approximately 9:00 pm, with an average concentration of 3.26 ppm, was produced by the traffic flows of the service sectors getting off work; and (d) the night-shift workers caused the first peak in the day at approximately 2:00 am, with an average concentration of 3.50 ppm.On the other hand, the data from the EPA monitoring station where located at an intersection nearby the sensor No. 3. The CO concentration ranges from 1.0 to 2.7 ppm. The temporal fluctuation shows a smoother pattern compared with the monitoring result of WSN but still captures the significant differences of air quality between rush-hour and regular traffic over different time of a diurnal cycle (as shown in Figure 7). The location of the EPA station is at the rooftop of a 4-meter-high building while the sensor from WSN is from a height of 1.5 m on lampposts and traffic signs. Therefore, it may be the reason that data from WSN shows more temporal fluctuations in a street-level environment and can capture more detailed ground information and risk of human exposure to traffic-related air pollution than an EPA station.

### 3.2. Street-Level Variations of Hourly Concentrations

**Figure 8:**
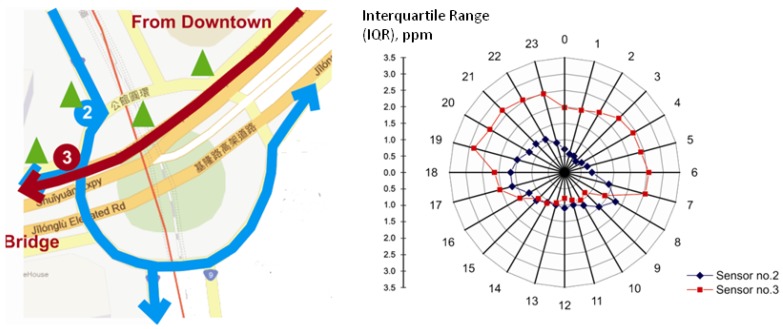
The hourly interquartile range (IQR) of CO concentrations of the sensor No. 2 and No. 3 at 24 hours a day from July to September 2010 (right panel). Sensor No. 3 represents a location affected by the difference of traffic flows on weekdays and weekends, but sensor No. 2 reflects a location that always accumulates high traffic volume from downtown to the residential areas (left panel). The traffic flows from downtown (blue lines) converge on the arterial and are diverted to different roads. The difference between weekdays and weekends only has a slight effect on sensor No. 2. The greater IQR of sensor No. 3 could be explained by the traffic flow (red line) only appearing during weekdays.

The hourly means show that the WSN captured detailed temporal dynamics. We could go further to explore the data variations of the different sensors to understand the traffic patterns in different locations. As shown in Figure 8, sensors No. 3 and No. 2 are selected to symbolize the difference of patterns between morning and evening. Sensor No. 3 is selected to represent a location that is affected by the difference of traffic flows on weekdays and weekends. Sensor No. 2 reflects a location that always accumulates high traffic volume from downtown to the residential areas. The difference between sensor No. 3 and No. 2 is that sensor No. 2 will not be influenced by the difference between weekdays and weekends. The arterial not only connects downtown to the satellite cities but also the southern part of the city. Traffic flows from downtown converge on the arterial and divert to roads R_a_, R_b_ and R_c_ every day. Therefore, the difference between weekdays and weekends only has a slight effect on sensor No. 2. The arterial where sensor No. 3 is located only address the traffic flows from downtown to the satellite cities by bridge. Hence, the traffic flows will present a clear difference between weekdays and weekends. We select sensors No. 2 and No. 3 to discuss the effect of weekends.

Interquartile range (IQR) of hourly data in this study, the range between Q_1_ and Q_3__,_ is used as the indicator for measuring the variations of the hourly CO concentrations of the sensors. Figure 8 depicts the hourly IQR of CO concentrations of the sensors from July to September 2010. A higher IQR in some hours indicates higher variations of concentrations in that hour of the study period. Being located near an arterial and between two traffic signs, the IQR of sensor No. 3 peaks at rush hour and diminishes during midday. This sensor shows a nearly triple IQR during rush hour than during midday. During the 6:00 am to 8:00 am rush hour, the CO concentration ranges from 1.00 to 3.35 ppm (IQR = 2.16). During 9:00 am to 2:00 pm, the CO concentration of sensor No. 2 ranges from 1.00 to 2.44 ppm (Average IQR = 1.02). It is higher than sensor No. 3 at 1 ppm but is lower than sensor No. 3 during rush hour. Sensor No. 2 presents two peaks during rush hour (8:00 am to 10:00 am and 6:00 pm to 8:00 pm) of approximately 1.50 to 1.70 ppm. However, the peaks of sensor No. 2 are still lower than those of sensor No. 3 at the same time.

Being only 60 m from sensor No. 3, the possible reason for the IQR difference may be its location. Located near a bus stop and just under a traffic sign, the arterial near sensor No. 2 is the only road that accumulates traffic flows from downtown, and traffic flows will be dispersed and diverted to satellite cities or residential areas south of the city under the traffic circle. As stated above, the traffic flows would not have a significant fluctuation between weekdays and weekends. Therefore, a smaller variance is recorded during midday. The greater IQR of sensor No. 3 could be explained by the different traffic patterns between weekdays and weekends. Due to the connection between downtown and the satellite cities, traffic congestion occurs due to commuting in the morning and evening on weekdays. Traffic congestion will increase the possibility of the interruption of automobiles by traffic signs. Therefore, the emission of CO is also increased by incomplete combustion. During the weekend, the traffic flow decreases to a lower level due to most of the labor force resting at home or taking trips outdoors. The high concentration during weekdays and the low readings from weekends worked together to produce variations of peaks in the timing of getting on and off work. However, the similar traffic flows at midday between weekdays and weekends resulted in lower variations at sensor No. 3.

There is a relatively higher IQR during midnight (11:00 pm to 5:00 am) at sensor No. 3. This sensor is located near the bridge connecting downtown and the satellite cities. The source of traffic flows consists not only of commuter vehicles but also freight vehicles. Freight vehicles such as trucks could tend to pass the bridge at midnight during weekdays to avoid traffic congestion, causing sensor No. 3 to have a higher IQR during midnight. Therefore, the difference of variations in hourly concentrations between sensor No. 2 and sensor No. 3 could reflect the difference of local environments subject to human activities.

### 3.3. Mapping Spatial-Temporal Variations of Hourly Concentrations

**Figure 9:**
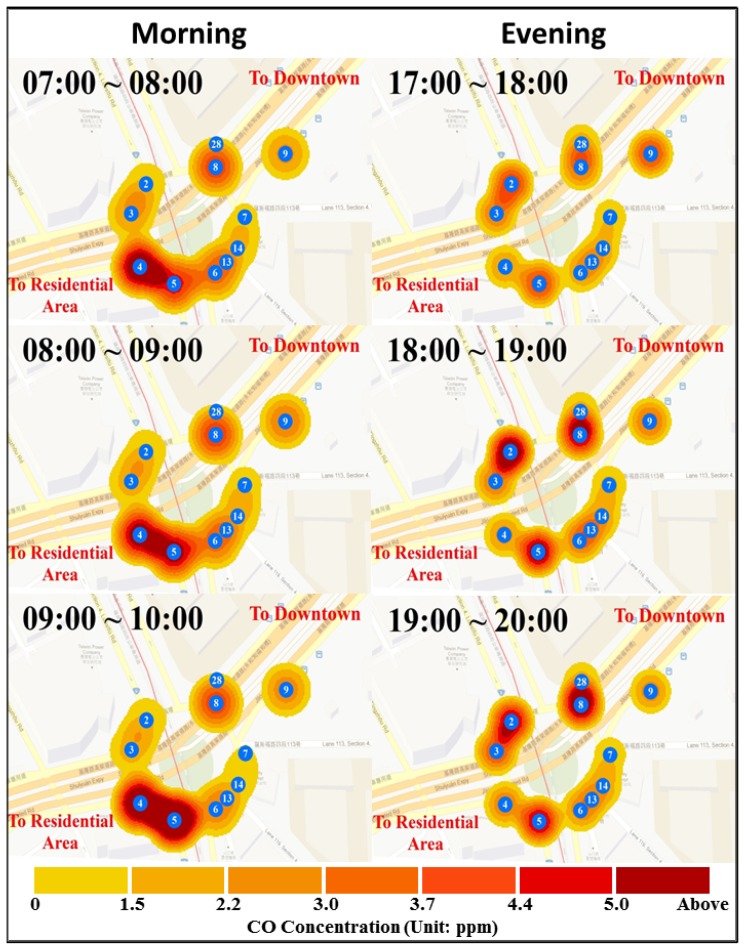
Street-level spatial-temporal variations around the circle: a clear on/off-peak traffic pattern is captured by WSN. In the morning, many vehicles drive from residential to downtown areas for work. This congestion induces a high concentration of air pollutants near a traffic signal. In the evening, the traffic flow to residential areas increases and it results in high concentrations at the contrast side.

The above discussions have shown that the CO concentration not only undulates with human activities but also presents variations of the traffic pattern in the micro-scale environment. It is proven that the WSN has the ability to capture detailed street-level fluctuations more effectively than existing approaches. We can go further to organize a spatial-temporal visualization, as shown in Figure 9, which compares the different concentration patterns between morning and evening. The congestion caused by morning/evening traffic flows from was selected as the study periods. The left side of Figure 9 shows the dynamics of the CO concentration from 7:00 am to 10:00 am, and the right side shows the dynamics from 6:00 pm to 8:00 pm.

In the morning, all sensors maintain low readings except sensor No. 4, which shows a CO concentration above 5 ppm from 7:00 am to 8:00 am. The concentration rises in sensor No. 5 progressively from 8:00 am to 9:00 am. There is only slight growth in sensor No. 9. The pattern of 9:00 am to 10:00 am shows a hotspot (above 5 ppm) in the CO concentration around sensors No. 4 and No. 5. In the evening, it seems there is a uniform distribution of CO concentrations around the circle during 6:00 pm. However, the concentration gradually grows in sensors No. 2 and No. 8 from 6:00 pm to 7:00 pm up to 5 ppm. The distribution maintains a similar pattern throughout 8 pm.

There is also a clear pattern that the CO concentration is overall more intensive in morning than evening. This result could be related to the special industrial culture within Asian countries such as Japan, Korea and Taiwan [29]. One of possible reasons could be that a salaried employee may clock in at approximately 8:00 am to 9:00 am, but they could not follow the same rule when getting off. Usually, a salaried employee can only leave the company after finishing their work. The traffic flows could be more disperse during evening than morning, therefore producing a higher and longer-lasting CO concentration from 7:00 am to 9:00 am. 

Previous studies have shown a positive correlation between traffic flows and the concentration of air pollutants such as CO [30,31,32]. The regularity of traffic flows will cause the CO concentration fluctuations. Certain regular cycles could be observed for all individuals 24 h a day. A person engages in a series of routine activities, such as sleeping, commuting and working following. From this perspective, because traffic flows is a representation of human activities, it seems that regularity plays an important role in the fluctuation of the CO concentration. Moreover, exposure to air pollutants affects public health in a significant way, especially in the street-level environment [33,34]. Heavy traffic contributes to most of the air pollutants on a road network. Exhaust containing CO is emitted when a vehicle engine transitions from idling to operation mode. Vehicles will have a higher possibility of stopping multiple times due to traffic signs. Therefore, serious traffic congestion exacerbates CO emission repeatedly due to a slower speed during heavy traffic periods. In our study, 7 of 11 (63.6%) sensors were distributed around a circle, revealing a pattern similar to that in Figure 7, which implies that the sensors located near the bus stop and pedestrian crossing monitored a similar pattern as sensor No. 3. People standing near the pedestrian crossing or bus stop around the circle will be exposed to higher concentrations of CO during rush hour. Therefore, evaluating the potential of wireless sensor networks to monitor the patterns would enhance our understanding of human exposure to air pollutants under the street-level environment. Furthermore, the dynamics of air pollutants from the traffic is a continuous space-time process. In further studies on space-time diffusion of air pollutants, not only the spatial-temporal variations of CO concentration can be monitored and analyzed separately, but sensor data from WSN-based framework would be beneficial to implement advanced spatial-temporal models for predicting the trends of high CO concentration corresponding to traffic conditions.

Although there are still some research limitations, there are some important implications from this study. First, in our analysis, we did not use a CO standard gas to calibrate the CO concentrations of the sensor. Instead, we calibrated the sensor by using real-time data from EPA station at the same time. The reason is that the micro-environmental conditions could have curtain effects on variations of CO readings. Therefore, before the deployment, we calibrated the CO concentrations of each wireless sensor by using data from the EPA station at the same time. Second, due to the accuracy and performance of the low-cost wireless sensors are not as good as the readings from EPA stations. Therefore, in this study the WSN-based framework is not proposed to replace the existing official monitoring systems. The purpose of the WSN-based monitoring framework plays a different role from the EPA stations. A wireless sensor may not provide high accuracy on measuring CO concentrations, but deploying large amount of spatially distributed sensors could provide significant spatial-temporal patterns in micro-scale environment [20,21]. The WSN-based framework could detect possible exceptional conditions and provide critical early warning signals for environment monitoring in time and space. Third, according to the Air Quality Standards by EPA of Taiwan and National Ambient Air Quality Standards (NAAQS) by the US EPA, the one-hour averaged CO concentration exceeding 35 ppm is considered harmful to public health and the environment. In our study the results showed that traffic congestion could cause an hourly average concentration of 5.3 ppm, which is lower than the air quality standard. However, the standard of CO concentration may not be appropriate as the early-warning threshold for street-level traffic pollutants, because human exposures to heavy traffic could be daily routine and long-term continually and chronically accumulated in urban areas [25]. Therefore, accumulated effects of traffic congestion should be further considered when EPA set the early-warning thresholds of CO concentration for traffic pollutants.

## 4. Conclusions

To understand the street-level variations of CO concentrations in an urban environment, this study proposed a pilot WSN-based framework with low power consumption for long-term environmental monitoring. Compared with traditional approaches employed by official environmental agencies, the WSN-based framework demonstrated its ability to monitor more-detailed variations of air pollutants in time and space. We differentiated the hourly fluctuations of CO between weekdays and weekends and compared the spatial-temporal patterns of air quality between rush-hour and regular traffic within one day to capture possible human behaviors and potential risk of human exposure to traffic-related air pollution. The proposed WSN-based framework provides street-level insights into real-time air quality monitoring for further early warning of air pollution and for urban environmental management.
